# Low let‐7d exosomes from pulmonary vascular endothelial cells drive lung pericyte fibrosis through the TGFβRI/FoxM1/Smad/β‐catenin pathway

**DOI:** 10.1111/jcmm.15989

**Published:** 2020-11-12

**Authors:** Han Xie, Yuan‐Mei Gao, Yong‐Chang Zhang, Ming‐Wang Jia, Fang Peng, Qing‐He Meng, Yi‐Chun Wang

**Affiliations:** ^1^ Department of Critical Care Medicine The Third Affiliated Hospital of Guangzhou Medical University Guangzhou China; ^2^ Department of Lung Cancer and Gastroenterology Hunan Cancer Hospital Changsha China; ^3^ Department of Surgery SUNY Upstate Medical University Syracuse U.S.A

**Keywords:** exosomes, FoxM1, let‐7d, lung pericyte fibrosis, TGFβRI, β‐catenin

## Abstract

The pathogenesis of pulmonary fibrosis (PF) was mediated by the progressive deposition of excessive extracellular matrix, but little is known about the regulatory mechanisms of fibrogenesis by lung pericytes. The mouse PF model was established by treatment with bleomycin, followed by isolation of exosomes from mouse broncho‐alveolar lavage fluids by the centrifuge method. Relative mRNA/microRNA levels and protein expression were assessed by qRT‐PCR and Western blotting, respectively. The binding of let‐7d with gene promoter was validated by dual‐luciferase reporter assay. Protein interactions were verified via GST pull‐down and co‐immunoprecipitation. Nuclear retention of Smad3 was analysed by extraction of cytoplasmic and nuclear fraction of pericytes followed by Western blotting. Association of FoxM1 with gene promoter was detected by EMSA and ChIP‐PCR methods. FoxM1 expression is significantly elevated in human lung fibroblasts of PF patients and mouse PF model. The expression of let‐7d is repressed in exosomes derived from broncho‐alveolar lavage fluids of PF mice. Let‐7d or FoxM1 knockdown suppressed the expression of FoxM1, Smad3, β‐catenin, Col1A and α‐SMA expression in mouse lung pericytes under TGF‐β1 treatment. FoxM1 overexpression elevated above gene expression in mouse lung pericytes under TGF‐β1 treatment. Let‐7d directly targets TGFβRI to regulate FoxM1 and downstream gene expression in mouse lung pericytes. FoxM1 directly interacts with Smad3 proteins to promote Smad3 nuclear retention and binds with β‐catenin promoter sequence to promote fibrogenesis. Exosomes with low let‐7d from pulmonary vascular endothelial cells drive lung pericyte fibrosis through activating the TGFβRI/FoxM1/Smad/β‐catenin signalling pathway.

## 1 INTRODUCTION

Pulmonary fibrosis (PF) is one severe interstitial lung disease featured by progressive deposition of excessive extracellular matrix (ECM) and lung tissue destruction with poor prognosis.^1^, ^2^, ^3^ Based on ethology, PF could be categorized into the most common idiopathic pulmonary fibrosis (IPF) with no known causes and other subtypes caused by other disorders such as rheumatoid arthritis and scleroderma, genetic mutations or exposure to radiation and hazardous chemicals.^2^, ^4^ The initiation and progression of PF have been known to be mediated by the alveolar epithelial cell activation, release of fibrogenic growth factors such as cytokines and growth factors, myofibroblast proliferation and activation, which resulted in abnormal ECM deposition and alterations.^2^ Nevertheless, the pathogenic mechanisms driving PF development are still far from being fully elucidated, which greatly hindered developing new therapeutic drugs for PF patients.

Pulmonary microvascular pericytes are responsible for generating collagen to maintain vascular stability in pulmonary vascular tissues by interacting with the epithelial cells under normal conditions.^5^ However, pulmonary microvascular pericytes could migrate to the pulmonary interstitium and transform into myofibroblasts expressing collagen, which has substantially contributed to the pathogenesis of PF.^5^, ^6^, ^7^ Recent investigations have demonstrated that TGF‐β1 (transforming growth factor β1) and its receptor TGF‐βRI (TGF‐β receptor I) promoted the transformation of pericytes into myofibroblasts during PF pathogenesis, mediated by the activation of Smad2/3 (Sma and Mad homologue 2/3) signalling pathway.^7^ In addition, the β‐catenin expression could also be up‐regulated by TGF‐β1 during the differentiation of lung pericytes into myofibroblasts.^8^ The activated Smad3 interacts with β‐catenin to promote the expression of fibrosis iconic proteins including α‐SMA, vimentin and collagen I (Col‐I) during pericytes‐to‐myofibroblast transition and PF development.^9^ However, the mechanisms regulating the TGF‐β1/Smad3/β‐catenin axis in PF pathogenesis remain poorly understood.

FoxM1 (Forkhead box M1) was originally characterized as a transcription factor regulating the expression of multiple functional genes associated with cell cycle progression.^10^, ^11^ Subsequent investigations revealed that FoxM1 served as a critical modulator of multiple other cellular processes such as cell proliferation and differentiation, apoptosis and DNA damage repair, thus promoting pathogenesis of human cancers and other disorders.^10^, ^12^ For instance, FoxM1 was identified as one downstream player of the Wnt/β‐catenin signalling which directly binds with β‐catenin to promote its nuclear localization and enhances the transcription factor activity of β‐catenin during glioma development.^13^, ^14^ Also, FoxM1 could directly promote the transcription of β‐catenin to activate the re‐annealing of endothelial adherent junctions during the repair of the injured vascular intima.^15^ In breast cancer cells, TIF1γ (transcriptional intermediary factor 1) is an E3 ubiquitin‐protein ligase responsible for the ubiquitination‐mediated degradation of Smad3/Smad4 protein complex, and FoxM1 protein could directly interact with the Smad3 proteins in the nucleus to inhibit the TIF1γ‐mediated Smad4 protein degradation and maintain the sustained activation of the Smad3/Smad4 complex.^16^ Importantly, recent report showed that the elevated FoxM1 was required for the differentiation of pericytes into myofibroblasts in TGF‐β1‐elicited PF.^17^ However, little is known about the roles of FoxM1 and its interaction with the TGF‐β1/Smad3/β‐catenin signalling in pulmonary microvascular pericytes during PF pathogenesis.

Exosomes are a large group of critical membrane vesicles with a common diameter of 40 to 100 nanometres, which could be secreted by various cell types such as cancer cells and epithelial cells.^18^, ^19^ Biochemically, exosomes are usually composed of multiple components including proteins, lipids, RNAs and small metabolites.^18^, ^20^ Although being originally considered as unwanted cell components, research in recent decades revealed that exosomes act as essential mediators of cellular signalling and intercellular communication because of their capability of carrying various bioactive macromolecules such as non‐coding RNAs, which was substantially involved in pleiotropic biological and pathogenic processes.^21^, ^22^ For instance, the miR‐10b, which was highly expressed in breast cancer cells, could be secreted into extracellular region in exosomes to enhance the metastasis capacity of cancer cells.^23^ The let‐7 microRNA (miRNA) family was recently found to repress the pathogenesis of IPF associated with sex steroid hormones.^24^ Also, let‐7d is one member of the let‐7 miRNA family and could inhibit the TGF‐β1‐induced epithelial‐to‐mesenchymal transition (EMT) during renal fibrogenesis by modulating the expression of signalling components downstream of TGF.^25^ However, the roles of let‐7d as well as its secretion in exosomes during PF development remain unclear.

In the present study, we aimed to test our hypothesis that exosomes carrying let‐7d secreted by pulmonary vascular endothelial cells might improve pulmonary pericyte fibrosis, as well as the possible mechanism mediated by the TGFβRI/FoxM1/Smad3/β‐catenin signalling cascade. These results would characterize new biomarkers associated with PF pathogenesis and provide a basis for developing new anti‐pulmonary fibrosis drugs.

## 2 MATERIALS AND METHODS

### 2.1 Clinical tissues and animal model

Human lung tissues were surgically collected from seven IPF patients registered to the Third Affiliated Hospital of Guangzhou Medical University (Guangzhou, China) and Hunan Cancer Hospital (Changsha,China) between December 2017 and March 2018. Lung tissues collected from seven healthy volunteers were used as negative control. The research was approved by the Medical Ethics Committee of the Third Affiliated Hospital of Guangzhou Medical University, and written consents were assigned by each participant before the surgery. The mouse pulmonary fibrosis model was established as previously introduced.^8^ Briefly, male C57/BL6 mice aged 8 weeks were purchased from Hunan SJA laboratory animal CO., LTD (Changsha, China) and sustained at 24 ± 2°C in autoclaved mouse cages with a 12‐h light/dark cycle. Following anaesthetization with ketamine (25 mg/kg) dissolved in xylazine, mice were intratracheally injected with bleomycin sulphate solution (1.5 U/kg bodyweight) and killed for lung tissue collection and other assays 21 days later. Mice injected with the same volume of normal saline were used as the control group. The animal model establishment was approved by the Experimental Animal Care and Use Committee of the Third Affiliated Hospital of Guangzhou Medical University.

### 2.2 Cell isolation and culture

The mouse lung microvascular epithelial line and mouse lung pericyte cell line were purchased from PriCells (Wuhan, China) and cultured in DMEM (Dulbecco's modified Eagle's medium) with supplementation of 10% foetal bovine serum (FBS) at 37°C in a humidified culture chamber with 5% CO_2_. The mouse lung fibroblasts and human lung fibroblasts were isolated from the lung tissues collected from model mice or IPF patients, respectively, as previously described.^8^ Briefly, the lung tissues collected from C57/BL6 mice after designated treatment or IPF patients were immersed in Hank's balanced salt solution followed by removal of vessel tissues and made into 1‐mm pieces. Tissue pieces were then digested with 0.25% trypsin (Sigma‐Aldrich) for 40 minutes, and the obtained cell suspension was filtered and centrifuged at 1000 rpm for 5 minutes. The precipitates were then resuspended in DMEM and centrifuged at 800 rpm for 5 minutes, and the resultant supernatant was centrifuged at 1000 rpm for another 5 minutes. Finally, the fibroblasts in the precipitate were collected and cultured in DMEM containing 10% FBS.

### 2.3 Cell transfection and treatment

For induction of fibrogenesis, mouse pulmonary microvascular pericytes were treated with TGF‐β1 (5 ng/mL; Sigma‐Aldrich) for 30 minutes at 37°C. To inhibit TGFβRI, cultured cells were treated with 3 μmol/L SB431542 (Sigma‐Aldrich) for 4 hours at 37°C. The shRNA targeting FoxM1, the let‐7d mimic, the let‐7d inhibitor, siRNA targeting Smad3 (siSmad3) and shβ‐catenin were synthesized by the GenePharma Company (Shanghai, China). For overexpression of FoxM1, the FoxM1 sequences amplified by RT‐PCR were ligated with the pcDNA3.0 plasmids to establish the pcDNA3.0‐FoxM1 recombinant plasmids. The above‐mentioned plasmids and sequences were transfected into cells using the Lipofectamine 3000 reagent (Thermo Fisher Scientific) following manufacturer's instructions.

### 2.4 Quantitative RT‐PCR

The relative expression of mRNA and microRNAs was detected by the quantitative RT‐PCR (qRT‐PCR) method using total RNA samples isolated from cells, exosomes or tissues with the TRIzol solution (#R0016; Beyotime, Beijing, China), following the manufacturer's instructions. Subsequently, the cDNA library was established from 2 μg RNA samples using the miScript II RT Kit (Cat. No. 218160; Qiagen) according to the manufacturer's instructions. The Talent Fluorescence Quantitative Detection Kit (SYBR Green) (#FP209; Tiangen Biotech, Beijing, China) was instructed by the manufacturer. The GAPDH was used as the internal standard, and relative expressional levels were finally calculated through the standard 2^‐△△Ct^ method based on at least three biological replicates.

### 2.5 Western blotting

Total protein samples were first exacted from cultured cells, exosomes or lung tissues using the RIPA Lysis and Extraction Buffer (#89900; Thermo Fisher Scientific) following the manufacturer's instructions. The protein concentration was determined by the BCA method. The separate purification of nuclear and cytosol proteins was accomplished using the Nuclear/Cytosol Fractionation Kit (#K266‐25; BioVision, USA) according to the manufacturer's instructions. Subsequently, 25 μg proteins of each group were boiled at 100°C for 5 minutes in protein loading buffer (#P0015; Beyotime, Beijing, China), separated by 10% or 12% SDS‐PAGE and transferred onto PVDF membrane (Millipore). The PVDF with proteins was then blocked with 5% BSA solution (Sangon Biotech, Shanghai, China), followed by incubation with primary antibodies and HRP‐conjugated secondary antibodies diluted in TBST. Finally, the expression of proteins was detected by developing with the enhanced chemiluminescence (ECL) reagents (#32106; Thermo Fisher Scientific), with GAPDH or β‐actin as the internal standard. Primary antibodies applied in this study include anti‐FoxM1 (#5436, human; CST), anti‐FoxM1 (ab180710, mouse; Abcam), anti‐TGF‐βRI (ab31013; Abcam), anti‐Smad3 (ab40854; Abcam), anti‐β‐catenin (#8480; CST), anti‐Col1A (ab34710; Abcam), anti‐α‐SMA (ab32575; Abcam), anti‐p‐Smad3 (#9520; CST), anti‐lamin B (ab16048; Abcam), anti‐Smad4 (#46535; CST), anti‐Flag (#14793; CST), anti‐Myc‐Tag (#2276; CST), anti‐TIF1γ (ab70560; Abcam), anti‐GAPDH (ab181602; Abcam) and anti‐β‐actin (ab8226; Abcam).

### 2.6 Histological evaluations

The immunohistochemistry (IHC) combined with haematoxylin and eosin (H&E) was performed to detect histological alteration and FoxM1 expression in lung tissues as previously introduced.^9^ Briefly, the lung tissues were fixed with 4% neutral phosphate‐buffered formalin for 2 hours at room temperature, embedded in paraffin and made into 5‐um slides. Subsequently, tissues slides were blocked with 5% BSA solution (Beyotime, Beijing, China), incubated with diluted primary antibodies targeting FoxM1 (ab180710; Abcam; 1:500) for 1 hour at room temperature, incubated with HRP‐conjugated secondary antibodies and developed with the DAB Substrate Kit (ab64238; Abcam), followed by staining with H&E. The histological alterations and protein expression in tissues were finally observed under microscopy. At least three biological repeats were accomplished.

### 2.7 Exosome isolation and characterization

Following the establishment of the mouse pulmonary fibrosis model, the broncho‐alveolar lavage fluids were collected from mice in the model group as well as the control group. The exosomes in broncho‐alveolar lavage fluids were isolated as introduced previously.^26^ The diameters of exosomes isolated from mouse broncho‐alveolar lavage fluids were then observed by electron microscopy (EM) method. Briefly, exosomes were fixed with 2% paraformaldehyde for 30 minutes at room temperature, loaded onto one copper‐formvar/carbon‐coated grids, followed by contrasting with 2% uranyl acetate, and finally observed by transmission electron microscope (JEOL 1010; 80 kV).

### 2.8 Dual‐luciferase reporter assay

To validate the direct binding of let‐7d with TGFβRI gene promoter region, the dual‐luciferase reporter assay was carried out using the pmirGLO Dual‐Luciferase miRNA Target Expression Vector System (Promega). The recombinant pmirGLO‐WT (wild‐type) TGFβRI and pmirGLO‐Mut (mutant) TGFβRI plasmids were established separately following the manufacturer's instructions, which were then transfected into the mouse lung microvessel pericytes using the Lipofectamine 3000 reagent (Thermo Fisher Scientific) as instructed by the manufacturer. Subsequently, cells transfected with different recombinant plasmids were transfected with the let‐7d mimic using the Lipofectamine 3000 reagent as well, followed by the detection of the luciferase activities using a GloMax^®^ 20/20 Luminometer (Promega).

### 2.9 Pull‐down and Co‐immunoprecipitation (Co‐IP)

For pull‐down assay, the coding sequences of the Smad3 gene were amplified by RT‐PCR and ligated with the pGEX‐4T‐1 vector, which was transferred into the E. coli expression strain, BL 21 (DE3). The recombinant GST‐Smad3 proteins were then isolated from BL 21 lysates using the glutathione Sepharose beads (#6555‐10; BioVision, USA) following the manufacturer's instructions. The 6X His‐tagged FoxM1 proteins were also expressed in BL 21 strain and purified using the Ni‐NTA agarose resin (Cat No. 30210; Qiagen) according to the manufacturer's instructions. The GST‐Smad3 proteins were incubated with His‐FoxM1, followed by pull‐down assay using the Pierce™ GST Protein Interaction Pull‐Down Kit (#21516; Thermo Fisher Scientific) as instructed by the manufacturer. After being washed with PBS, the collected resins were boiled in protein loading buffer and subjected to Western blotting using antibodies targeting FoxM1. The in vivo interaction between proteins was validated using the Pierce™ Co‐Immunoprecipitation Kit (#26149; Thermo Fisher Scientific) following the protocol provided by the manufacturer.

### 2.10 Electrophoretic mobility shift assay (EMSA)

The direct binding of FoxM1 protein with the promoter sequences of the β‐catenin gene was confirmed in vitro by EMSA using the LightShift™ Chemiluminescent EMSA Kit (#20148; Thermo Fisher Scientific) following the manufacturer's instructions. Briefly, the DNA target sequences of the β‐catenin gene used as the native probe, as well as the mutant probe, were biotinylated using the Biotin 3' End DNA Labeling Kit (#89818; Thermo Fisher Scientific) according to the manufacturer's instructions. Subsequently, the native probe was incubated with the FoxM1 proteins or in combination with anti‐FoxM1 antibodies, followed by Western blotting using primary antibodies targeting biotin. Also, the mutant probe incubated with the FoxM1 proteins was applied as the negative control.

### 2.11 Chromatin Immunoprecipitation (ChIP)

The validation of FoxM1 protein binding with β‐catenin gene promoter region in mouse lung pericytes by ChIP was finished using the ChIP Kit (#ab500; Abcam) following protocol provided by the manufacturer. Briefly, the cultured lung pericytes were first fixed with the mixture of formaldehyde, 1 × Buffer A and PBS for 12 minutes at room temperature, followed by incubation with the Buffer B for 15 minutes at room temperature, incubation with Buffer C for 12 minutes at room temperature and incubation with the mixture of Buffer D and PI for 15 minutes at room temperature. The optimal DNA fragmentation was induced by appropriate sonication of the above cell mixture, which was subjected to centrifuge at 14 000 *g* for 10 minutes at 4°C. The resultant supernatant containing DNA fragments was immunoprecipitated with the antibodies targeting the FoxM1 proteins, and the bound DNAs were subsequently purified using the DNA purifying slurry. Finally, the existence of β‐catenin gene promoter sequences of interest was detected by the PCR method. DNA fragments precipitated with anti‐IgG antibodies were used as the negative control.

### 2.12 Statistical analysis

The SPSS 22.0 software was used to test the significance of quantitative data presented as mean ± SD (standard deviation) based on at least three biological replicates. The differences between two or more groups were analysed by the Student's *t* test and the ANOVA (analysis of variance) methods, respectively. The significant differences were defined by *P* < .05.

## 3 RESULTS

### 3.1 Elevated FoxM1 expression in IPF patients and mouse models

**FIGURE 1:**
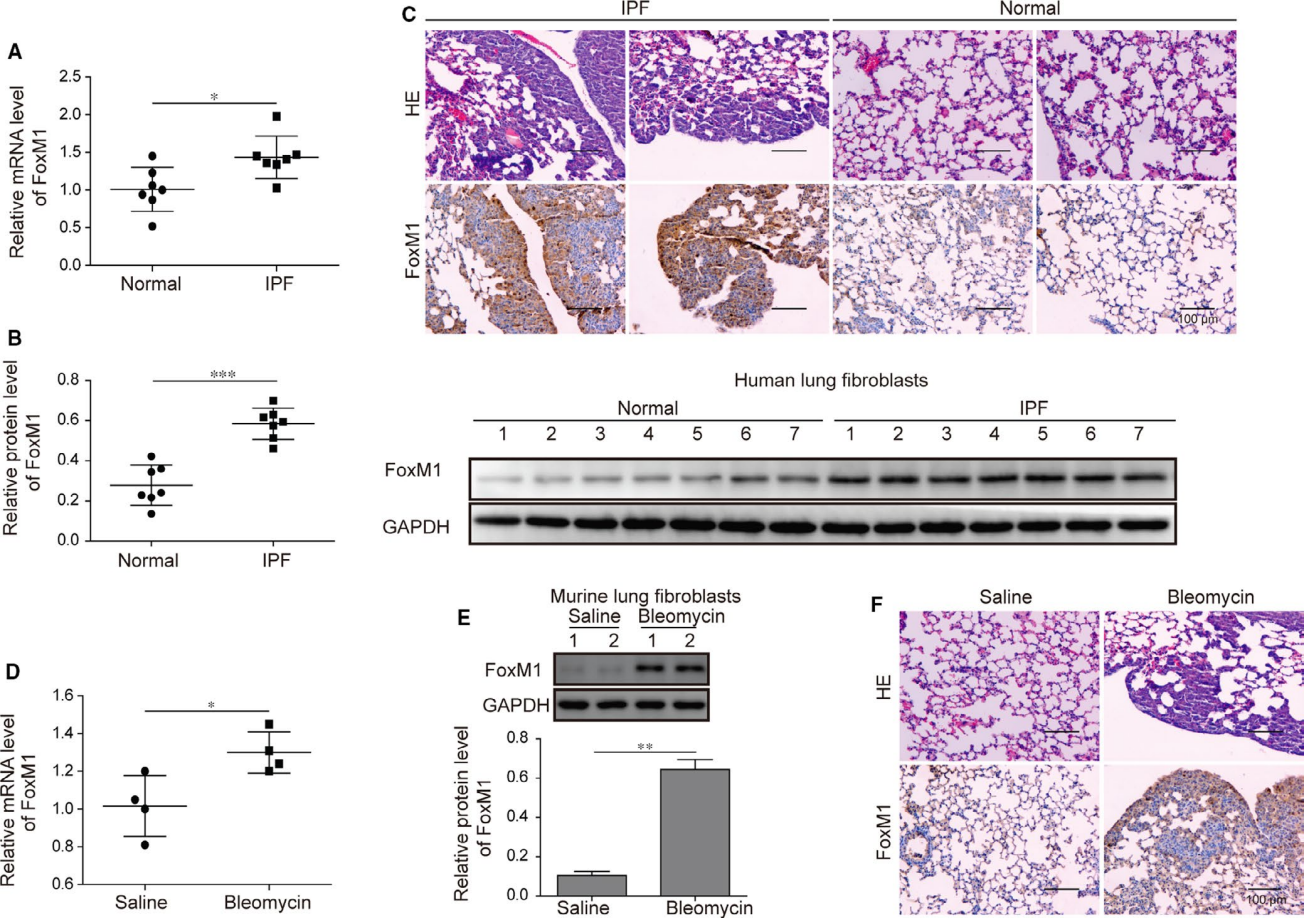
Significant increase in FoxM1 gene expression associated with pulmonary fibrosis. A and B, The expression of FoxM1 in lung fibroblasts isolated from lung tissues of IPF patients. FoxM1 mRNA and protein levels in fibroblasts were detected by qRT‐PCR (A) and Western blotting (B). C, Histological evaluations of lung tissues collected from IPF patients. The histological alteration of lung tissues was observed following H&E staining, and FoxM1 protein abundances in lung tissues were analysed by IHC assay. D and E, The increased expression level of FoxM1 in lung tissues from mouse pulmonary fibrosis model. The mice were treated with bleomycin sulphate solution (1.5 U/kg bodyweight) or the same volume of normal saline by intratracheal injection. FoxM1 mRNA and protein levels in mouse lung tissues were measured by qRT‐PCR (D) and Western blotting (E). F, Histological features and FoxM1 protein expression in the lung tissues of pulmonary fibrosis model mice. Lung tissues from mice were analysed by H&E and IHC assays. IPF: idiopathic pulmonary fibrosis; FoxM1: Forkhead box M1; GAPDH: glyceraldehyde‐3‐phosphate dehydrogenase; *P < .05; **P < .01

To investigate the potential involvement of FoxM1 during IPF pathogenesis, we first isolated the lung fibroblasts from clinical tissues collected from eight patients diagnosed with IPF, and fibroblasts from healthy volunteers were used as the negative group. Through qRT‐PCR, we showed that the FoxM1 mRNA levels in lung fibroblasts from IPF patients were significantly higher than those of the normal group (Figure 1A). Also, the increased expression of FoxM1 in lung fibroblasts isolated from IPF patients was further confirmed by our following Western blotting assay (Figure 1B). Moreover, we analysed the histological alterations of lung tissues in IPF patients and observed significant fibrosis and disordered structure in the lung tissues of IPF patients, in comparison with those of the normal group (Figure 1C). In consistence, we found by IHC analysis that the expression of FoxM1 proteins in the lung tissues of IPF patients was also greatly higher than that in those of the normal group (Figure 1C). Next, we established the mouse pulmonary fibrosis model by treatment with bleomycin as introduced in the Material and Methods section. Through qRT‐PCR, Western blotting and IHC methods, we showed that the FoxM1 expression in the lung tissues of mouse model was remarkably elevated compared with the control group (treated with saline; Figure 1D‐F). Significant tissue fibrosis was also observed in the lung tissues of the mouse model, but not in the control group (Figure 1F). These results showed the significantly increased FoxM1 expression in lung tissues during PF pathogenesis.

### 3.2 Decreased let‐7d in exosomes and increased FoxM1 expression from mouse PF model

**FIGURE 2:**
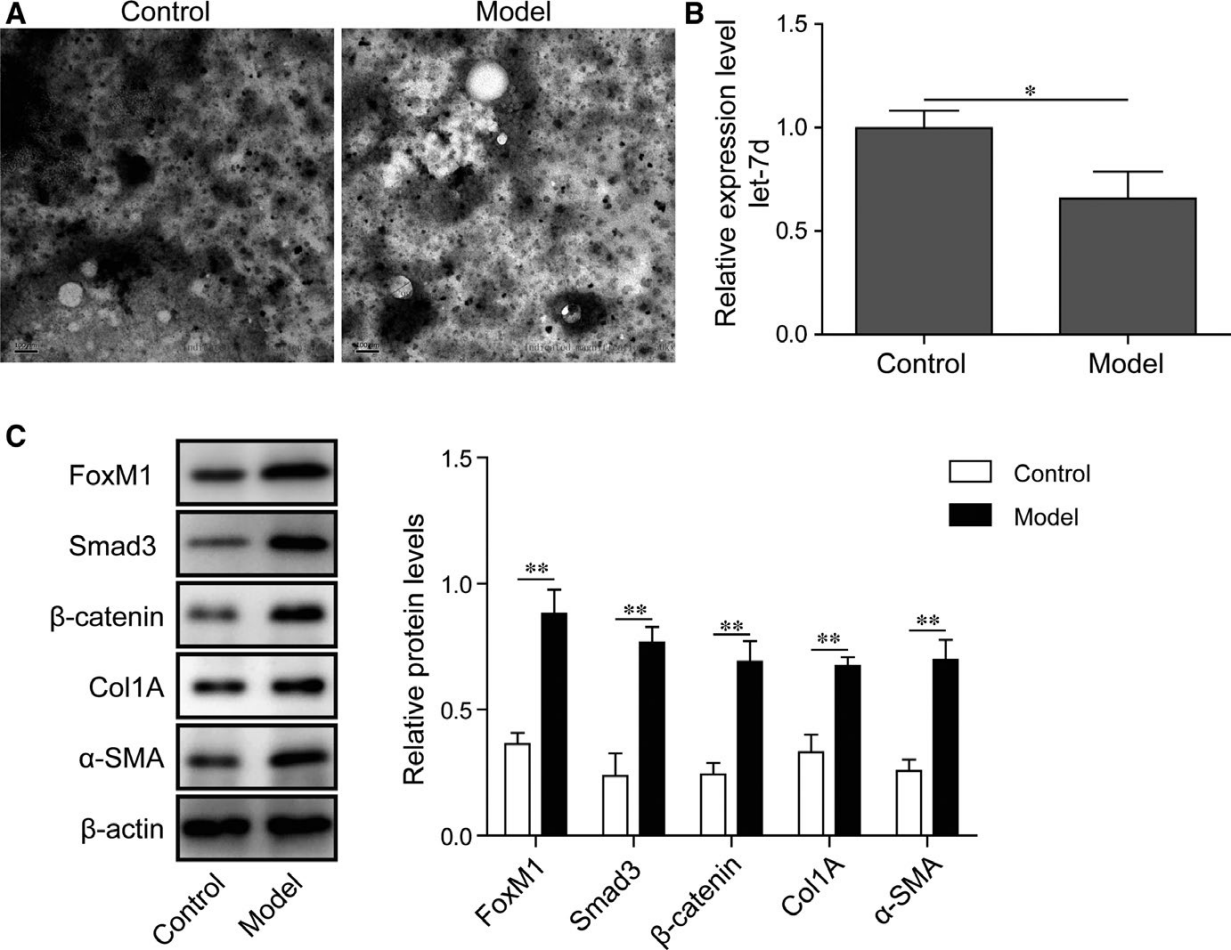
Decreased let‐7d in exosomes and increased FoxM1 expression from mouse PF model. A, Isolation of exosomes from broncho‐alveolar lavage fluids of the pulmonary fibrosis model mice. The PF model was established by intratracheal injection of bleomycin sulphate solution (1.5 U/kg bodyweight). Mice treated with the same volume of normal saline were used as the control. The morphology and diameters of isolated exosomes were observed by the transmission electron microscopy. B, The expression of let‐7d in exosomes isolated from broncho‐alveolar lavage fluids of pulmonary fibrosis model mice. RT‐qPCR method was performed to analyse let‐7d expression in exosomes. C, Changes of FoxM1, signalling components and fibrosis‐related genes in lung pericytes treated with exosomes from pulmonary fibrosis model. FoxM1, Smad3, β‐catenin, Col1A and α‐SMA protein levels were measured by Western blotting. FoxM1: Forkhead box M1; Smad3: Sma and Mad homologue 3; Col1A: collagen Iα; α‐SMA: α‐smooth muscle actin; *P < .05; **P < .01

For analysis of the roles of exosomes in pathogenesis of PF, we then isolated the exosomes from broncho‐alveolar lavage fluids of the mouse PF model as well as the normal control group, as described in the Material and Methods section. Our following TEM analysis showed that exosomes from mouse broncho‐alveolar lavage fluids had a diameter of 30‐100 nm, which was consistent with previous reports (Figure 2A). Also, we observed the expression of let‐7d in exosomes isolated from both the control group and PF model, and significant decrease of let‐7d expression in the model group compared with the control group (Figure 2B). On the contrary, our following Western blotting assay also showed that FoxM1 protein expression in mouse lung pericytes treated with exosomes from PF mice was markedly higher than that of the control group (Figure 2C). Furthermore, we also found that the expression of key signalling components and fibrosis‐related genes in mouse lung pericytes was greatly increased by exosomes from model mice compared with those treated with exosomes from the control group, including Smad3, β‐catenin, Col1A and α‐SMA (Figure 2C). These results indicated the roles of exosomes with low let‐7d and pericytes with high FoxM1 expression in regulating PF pathogenesis.

### 3.3 Let‐7d mitigated FoxM1‐promoted expression of Smad3, β‐catenin and fibrosis‐related genes in mouse lung pericytes under TGF‐β1 treatment

**FIGURE 3:**
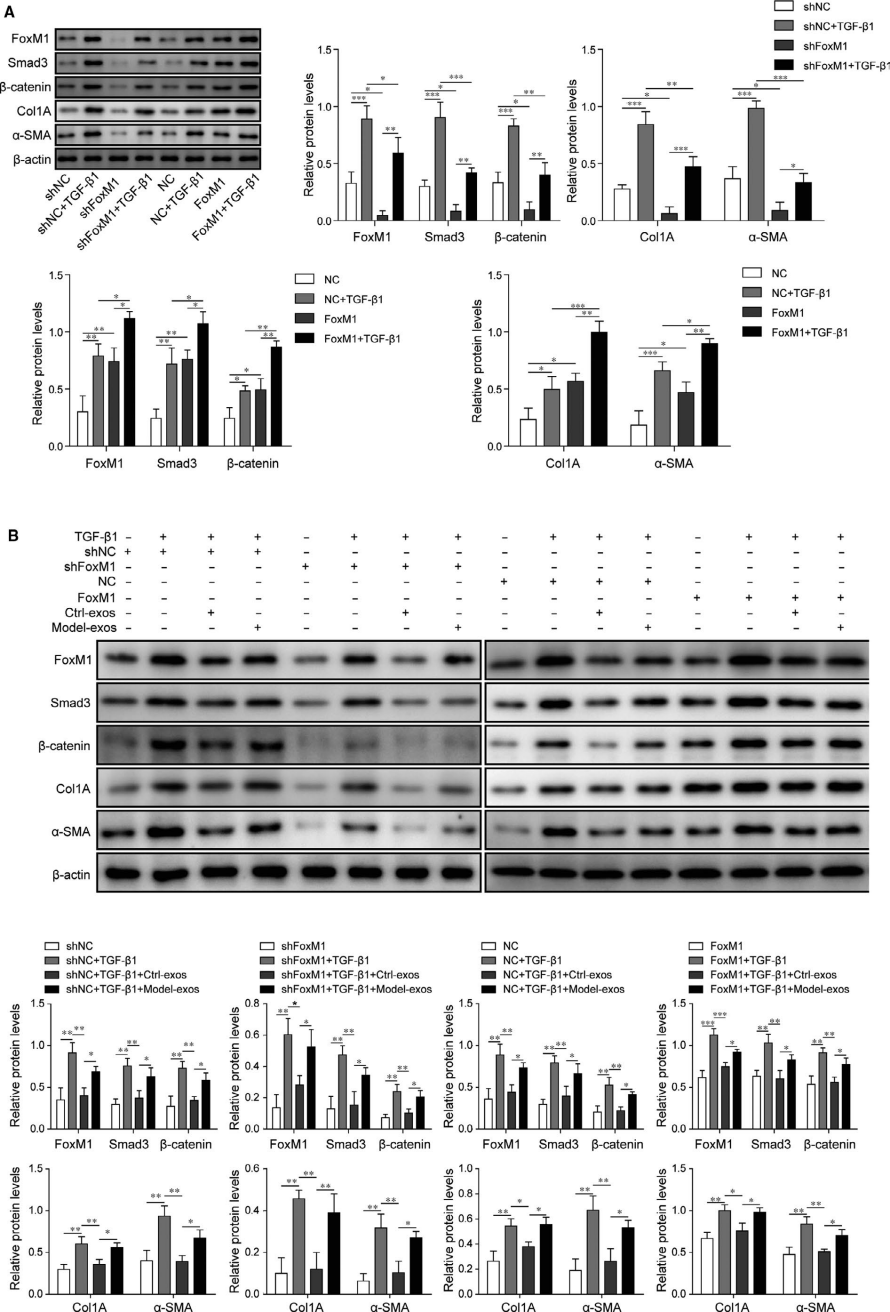
Let‐7d mitigated FoxM1‐promoted expression of Smad3, β‐catenin and fibrosis‐related genes in mouse lung pericytes under TGF‐β1 treatment. A, Mouse lung pericytes were treated with TGF‐β1 (5 ng/mL) for 1 h, in combination with FoxM1‐overexpressing plasmids or shFoxM1, and the abundances of FoxM1, Smad3, β‐catenin, Col1A and α‐SMA proteins in mouse pericytes under designated treatments were then detected by Western blotting. B, Mouse lung pericytes were treated with TGF‐β1 (5 ng/mL) for 1 h, in combination with FoxM1‐overexpressing plasmids, shFoxM1 or exosomes from control or model group (Ctrl‐exos or Model‐exos). The abundances of FoxM1, Smad3, β‐catenin, Col1A and α‐SMA proteins in mouse pericytes under designated treatments were then detected by Western blotting. TGF‐β1: transforming growth factor β1; FoxM1: Forkhead box M1; shFoxM1: shRNA targeting FoxM1; NC: negative control; Smad3: Sma and Mad homologue 3; Col1A: collagen Iα; α‐SMA: α‐smooth muscle actin; *P < .05; **P < .01; ***P < .001

To further investigate the effects of FoxM1 and let‐7d in regulating PF development, we then analysed the expression of FoxM1 and downstream signalling and fibrosis‐related genes in mouse pericytes treated with TGF‐β1 by Western blotting. We found that the TGF‐β1 treatment significantly elevated the protein levels of FoxM1 in mouse pericytes compared with the control group (Figure 3A). Also, the protein levels of Smad3, β‐catenin, Col1A and α‐SMA in mouse pericytes were all significantly increased by TGF‐β1 treatment compared with the control group (Figure 3A). Under TGF‐β1 treatment, the overexpression of FoxM1 caused much more significant increase of FoxM1 expression in mouse pericytes in comparison with the control group, as well as remarkable increases of Smad3, β‐catenin, Col1A and α‐SMA expression (Figure 3A). On the contrary, the knockdown of FoxM1 expression with specific shRNA targeting FoxM1 (shFoxM1) effectively suppressed the increases of FoxM1, Smad3, β‐catenin, Col1A and α‐SMA expression in mouse pericytes induced by TGF‐β1 treatment (Figure 3A). To assess the effect of exosomes derived from normal or PF model (Ctrl‐exos or Model‐exos) on FoxM1, Smad3, β‐catenin, Col1A and α‐SMA expression in pericytes, the primary cultured pericytes were treated with exosomes for 48 hours, and we found that the treatment with Model‐exos including low let‐7d remarkably up‐regulated the expression of FoxM1, Smad3, β‐catenin, Col1A and α‐SMA proteins in mouse pericytes under TGF‐β1 treatment or combined with FoxM1 overexpression or knockdown (Figure 3B). These results showed that the elevation of FoxM1 and downstream signalling component expression in mouse lung pericytes could be repressed by let‐7d.

### 3.4 Let‐7d targets TGFβRI to suppress FoxM1 and downstream gene expression in mouse lung pericytes

**FIGURE 4:**
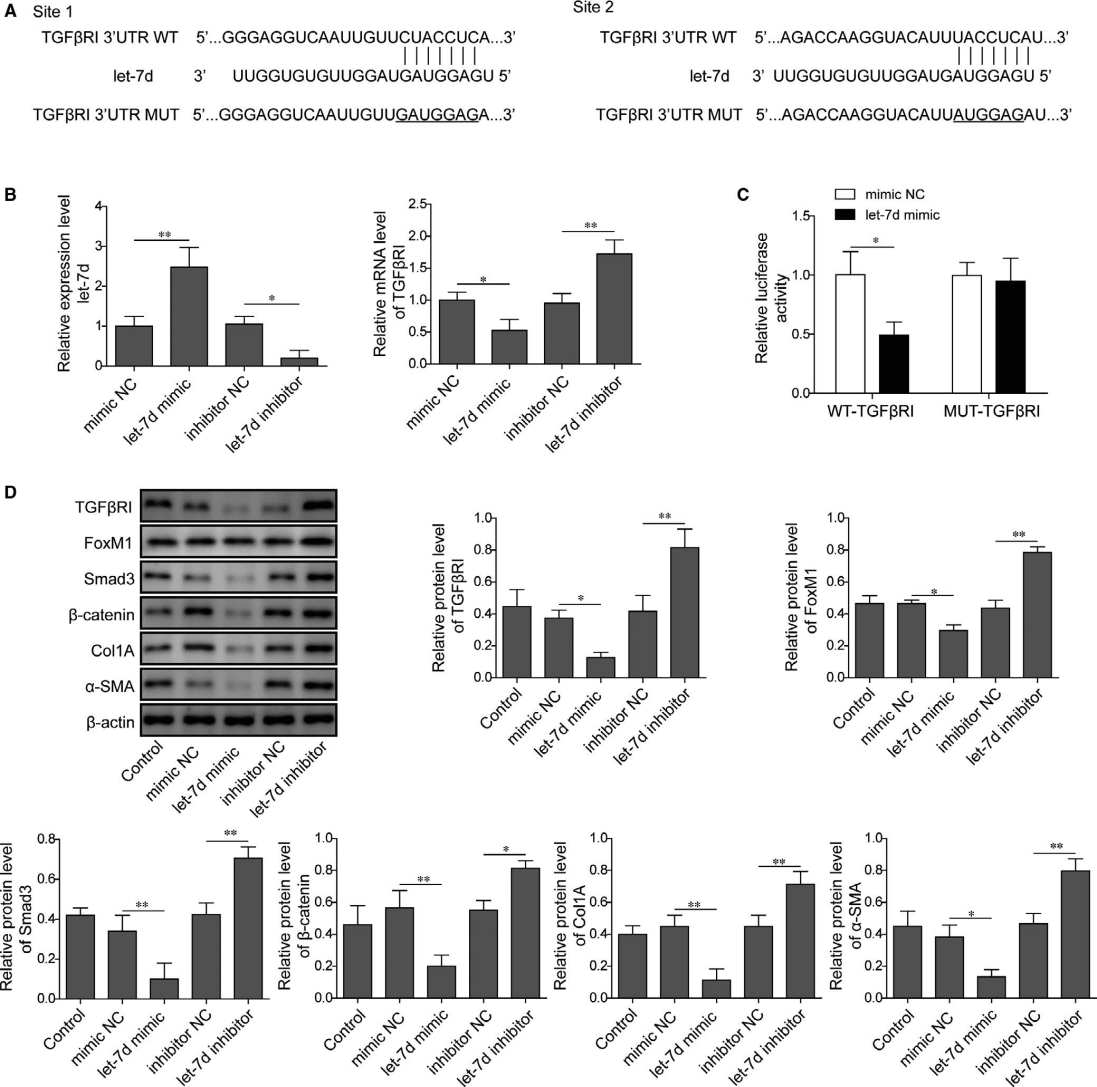
Let‐7d directly targets TGFβRI promoter to repress FoxM1 and fibrosis gene expression in mouse lung pericytes. A, The direct binding of let‐7d with the promoter regionuntranlational region of the TGFβRI shown by bioinformatic analysis. B, Relative expression level of let‐7d and TGFβRI in mouse lung pericytes treated with let‐7d mimic or inhibitor. Mouse lung pericytes were transfected with let‐7d mimic, inhibitor or their corresponding negative control sequences for 24 h. The relative mRNA levels were detected by qRT‐PCR method. C, Direct binding of let‐7d with the promoter region of the TGFβRI in mouse lung pericytes. The dual‐luciferase reporter assay was performed to test the association of let‐7d with TGFβRI 3'UTR sequences. D, The abundances of TGFβRI, FoxM1, Smad3, β‐catenin, Col1A and α‐SMA proteins in mouse lung pericytes treated with let‐7d mimic or inhibitor. Twenty‐four hours after the transfection with let‐7d mimic, inhibitor or their corresponding negative control sequences, protein levels in mouse lung pericytes were quantified by Western blotting. TGFβRI: transforming growth factor beta receptor I; 3'UTR: 3' untranslated region; NC: negative control; WT: wild‐type; MUT: mutant; FoxM1: Forkhead box M1; Smad3: Sma and Mad homologue 3; Col1A: collagen Iα; α‐SMA: α‐smooth muscle actin; *P < .05; **P < .01

For more insights into the downstream target of let‐7d in lung pericytes during PF development, we then performed a bioinformatic analysis and showed that let‐7d might directly bind with the promoter region of the TGFβRI (Figure 4A). Moreover, we found that treatment with let‐7d mimic significantly elevated let‐7d expression but suppressed TGFβRI expression in mouse pericytes compared with the negative control (NC) group (Figure 4B). Contrarily, the treatment with let‐7d inhibitor effectively repressed let‐7d expression in mouse pericytes, which caused greatly elevated expression of TGFβRI in mouse lung pericytes compared with the negative control group (Figure 4B). Through dual‐luciferase reporter assay, we showed that the let‐7d induced significant decrease of luciferase activity in mouse lung pericytes expressing the wild‐type TGFβRI 3' un‐translated region (3'UTR) sequences, but not in the pericytes expressing the mutant TGFβRI 3'UTR sequences, showing the direct binding of let‐7d with the 3'UTR of TGFβRI (Figure 4C). Subsequently, we showed that let‐7d mimic remarkably down‐regulated the expression of TGFβRI, FoxM1, Smad3, Col1A and α‐SMA in mouse lung pericytes compared with the NC group (Figure 4D). On the contrary, the let‐7d inhibitor caused significant increase of these genes in mouse lung pericytes in comparison with the NC group (Figure 4D). These results showed that let‐7d could suppress the expression of FoxM1 and downstream signalling and fibrosis‐related genes in lung pericytes by directly targeting TGFβRI.

### 3.5 FoxM1 interacts with Smad3 protein to promote nuclear retention of Smad3 proteins in lung pericytes

**FIGURE 5:**
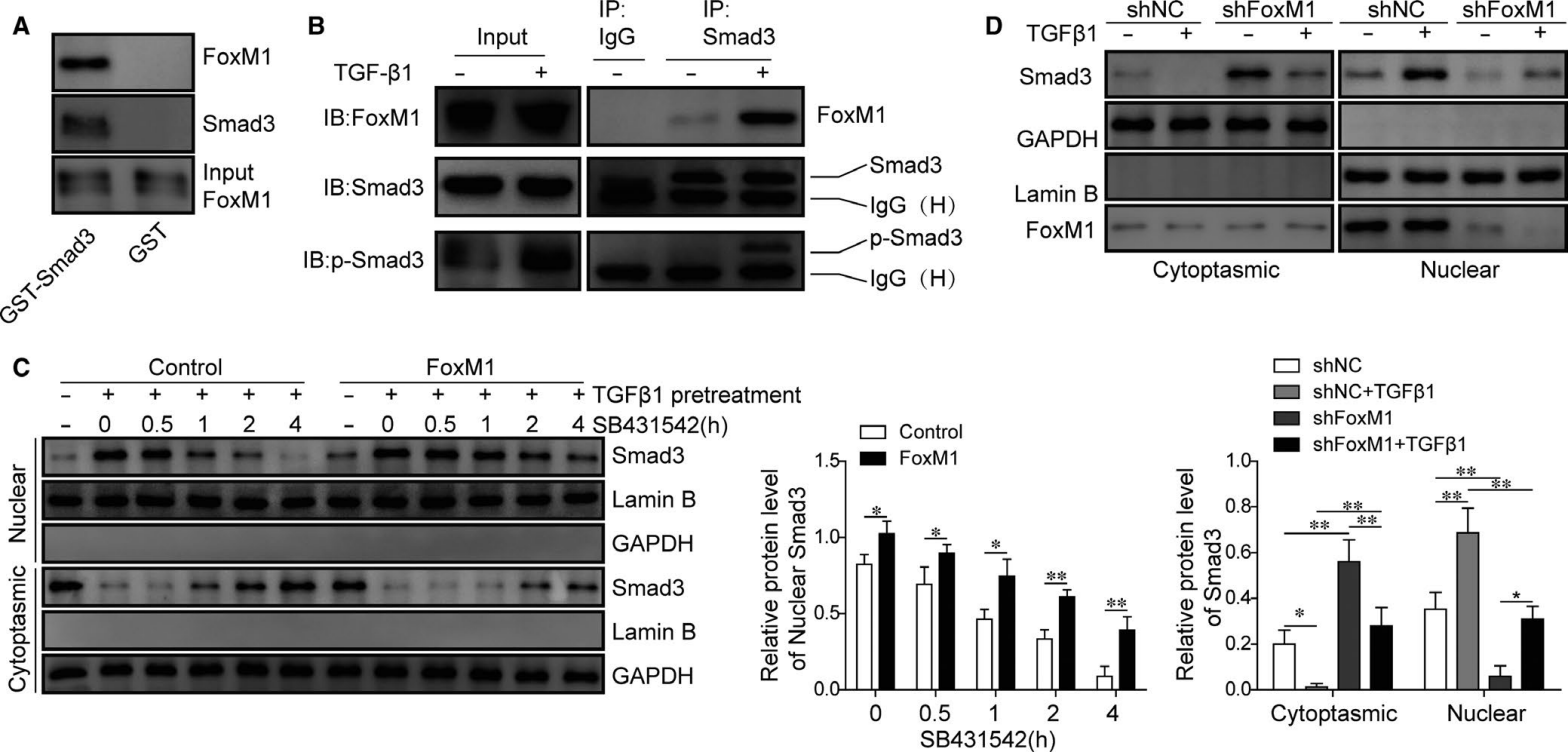
Enhanced Smad3 nuclear retention by its interaction with FoxM1 in mouse lung pericytes. A, The direct binding of recombinant 6X His‐FoxM1 proteins with recombinant GST‐Smad3 proteins confirmed by in vitro GST pull‐down assay. B, The in vivo association of endogenous FoxM1 proteins with Smad3 proteins in mouse lung pericytes treated with TGF‐β1. Lung pericytes were treated with TGF‐β1 (5 ng/mL) for 1 h and followed by the validation of protein interaction by Co‐IP assay. C, Relative Smad3 protein abundances in the cytoplasmic and nuclear fractions of FoxM1‐overexpressing mouse lung pericytes under treatments with TGF‐β1 and SB431542. Mouse lung pericytes were treated with 5 ng/mL TGF‐β1 for 1 h combined with 3 μmol/L SB431542 for 0.5 to 4 h as designated. Protein levels in cytoplasmic or nuclear factions were detected by Western blotting, using GAPDH as the internal standard and lamin B as the nuclear fraction marker. D, Effects of shFoxM1 on the nuclear retention of Smad3 and phosphorylated Smad3 proteins in mouse lung pericytes under TGF‐β1 treatment (5 ng/mL; 1 h). GST: glutathione S‐transferase; Smad3: Sma and Mad homologue 3; FoxM1: Forkhead box M1; TGF‐β1: transforming growth factor beta1; IP: immunoprecipitation; IB: immunoblotting; p‐Smad3: phosphorylated Smad3; shFoxM1: shRNA targeting FoxM1; GAPDH: glyceraldehyde‐3‐phosphate dehydrogenase; *P < .05; **P < .01

To study the molecular mechanisms of FoxM1 functioning in PF, we first test the possibility of direct interaction between FoxM1 and Smad3. Through pull‐down assay, we showed that FoxM1 protein could be effectively pulled down together with GST (glutathione S‐transferase)‐tagged Smad3 proteins, showing the direct interaction of FoxM1 proteins with the Smad3 proteins in vitro (Figure 5A). Our following Co‐IP assay also showed that endogenous FoxM1 protein could directly associate with Smad3 proteins in mouse lung pericytes (Figure 5B). Also, the treatment of TGF‐β1 effectively enhanced the association of endogenous FoxM1 proteins with Smad3 proteins in mouse lung pericytes, as well as the interaction of FoxM1 proteins with phosphorylated Smad3 proteins (Figure 5B). Through separate purification of the cytosol and nuclear fractions of mouse lung pericytes combined with Western blotting, we showed that TGF‐β1 treatment significantly increased the Smad3 protein levels in the nuclear fractions of mouse lung pericytes, together with decrease of Smad3 in cytoplasmic fractions of mouse lung pericytes treated with TGF‐β1 (Figure 5C). Moreover, we found that the Smad3 protein levels in nuclear fractions of mouse lung pericytes under TGF‐β1 treatment could be further elevated by overexpression of FoxM1 (Figure 5C). The nuclear Smad3 protein levels in mouse lung pericytes under TGF‐β1 treatment were also shown to be reduced by the treatment with TGFβRI inhibitor SB431542 in a concentration‐dependent manner (Figure 5C). Furthermore, we showed that the knockdown of FoxM1 with shFoxM1 significantly reduced the nuclear FoxM1 levels in mouse lung pericytes (Figure 5D). In addition, the increased nuclear Smad3 protein level in mouse lung pericytes under TGF‐β1 treatment was also greatly down‐regulated by shFoxM1 transfection (Figure 5D). These results showed that FoxM1 could directly associate with Smad3 proteins to promote the TGF‐β1‐induced nuclear retention of Smad3 proteins in mouse lung pericytes.

### 3.6 FoxM1 attenuates TIF1γ‐mediated Smad4 protein ubiquitination and TGF‐β1 signalling inhibition in mouse lung pericytes

**FIGURE 6:**
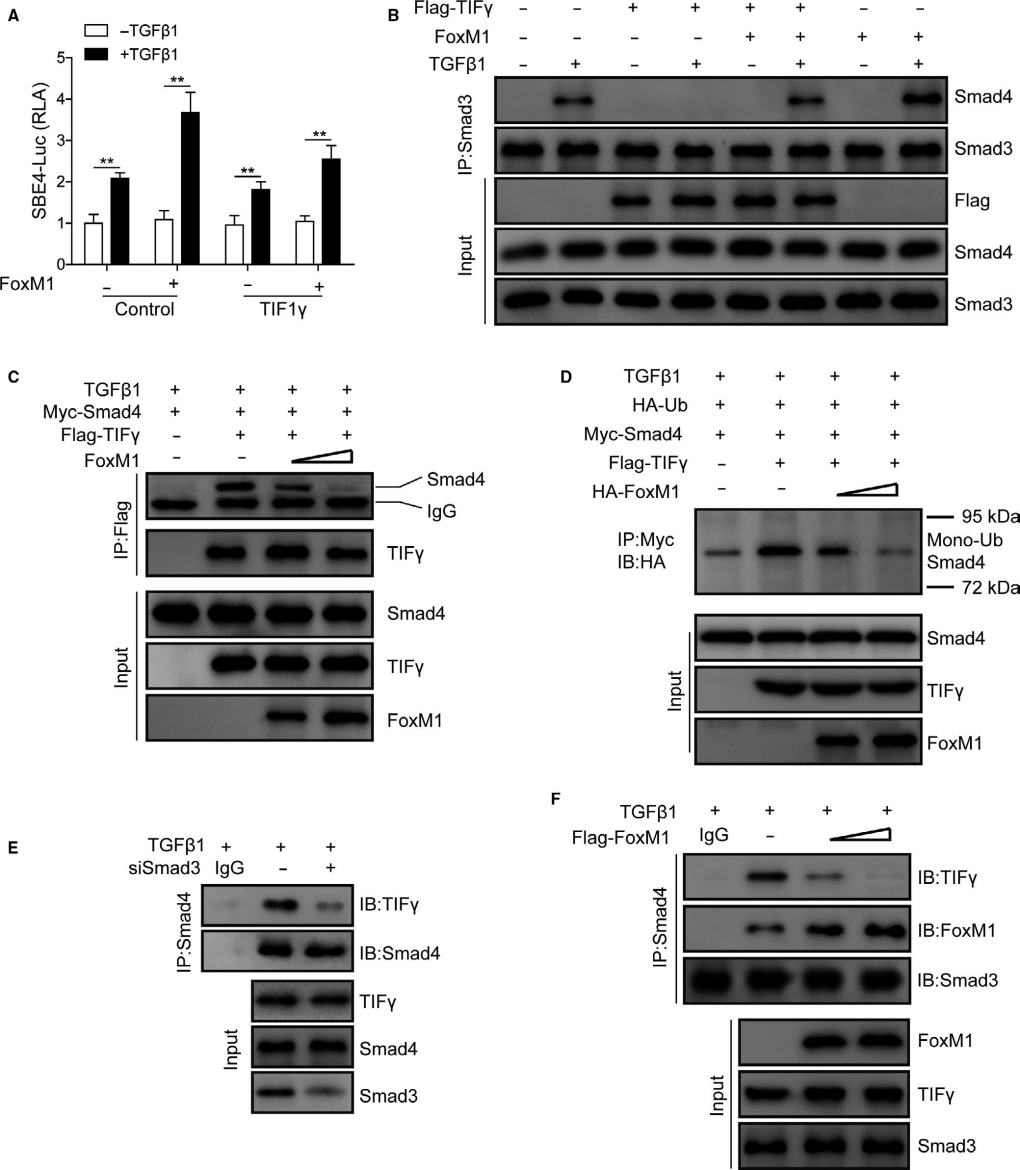
Suppression of TIF1γ‐induced Smad4 protein ubiquitination and deactivation of TGF‐β1 signalling by FoxM1 overexpression in lung pericytes. A, FoxM1 attenuated the inhibitory effect of TIF1γ on Smad3‐activated gene transcription. Pericytes with FoxM1 and TIF1γ overexpression were treated with TGF‐β1 (5 ng/mL) for 20 h following measurement of the luciferase assay. B, Abrogation of the TIF1γ‐induced inhibition of the formation of the Smad3/Smad4 complex in lung mouse pericytes by FoxM1 overexpression. Pericytes transfected with FoxM1‐ and TIF1γ‐expressing plasmids as indicated were harvested 48 h after TGF‐β1 treatment (5 ng/mL) for 2 h. The formation of the Smad3/4 protein complex was evaluated by co‐immunoprecipitation. C, Suppression of the exogenous association of Smad4 with TIF1γ in mouse lung pericytes by FoxM1 overexpression. Pericytes transfected with indicated plasmids were treated with TGF‐β1 (5 ng/mL) for 16 h, which were harvested for co‐immunoprecipitation. D, The inhibition of TIF1γ‐mediated Smad4 protein mono‐ubiquitination by FoxM1 overexpression in mouse lung pericytes. Following transfection with indicated plasmids, cells were treated with TGF‐β1 (5 ng/mL) for 16 h. E, Effects of Smad3 knockdown on the interaction between TIF1γ and Smad4 proteins in mouse lung pericytes. Cells transfected with Smad3 siRNAs or negative control were subjected to TGF‐β1 (5 ng/mL) treatment for 2 h and harvested 48 h later. F, Inhibition of the association of Smad3 with TIF1γ by FoxM1 overexpression in mouse lung pericytes. Cells transfected with the indicated plasmids were treated with 5 ng/mL TGF‐β1 for 2 h. TGF‐β1: transforming growth factor beta1; FoxM1: Forkhead box M1; TIF1γ: transcriptional intermediary factor 1 γ; IP: immunoprecipitation; IB: immunoblotting; Smad3/4: Sma and Mad homologue 3/4; *P < .05; **P < .01

To further investigate the molecular events downstream of FoxM1 in PF pathogenesis, we then analysed the involvement of TIF1γ‐induced inhibition of Smad3/4 functions as transcription factor. Using the Smad4‐responsive SBE4‐Luc reporter gene, we found that TGF‐β1 treatment enhanced the luciferase activity of the SBE4‐Luc reporter gene in mouse lung pericyte, which could be significantly inhibited by the overexpression of the TIF1γ, showing the inhibition of TIF1γ on the Smad3/4‐mediated downstream gene transcription (Figure 6A). However, the inhibition of Smad3/4‐mediated transcription by TIF1γ in mouse lung pericytes under TGF‐β1 treatment could be partially attenuated by FoxM1 overexpression (Figure 6A). Also, the formation of Smad3/4 protein complex in lung pericytes caused by TGF‐β1 treatment could be inhibited by TIF1γ overexpression, but FoxM1 overexpression significantly relieved the inhibition of formation of Smad3/4 protein complex by TIF1γ (Figure 6B). Moreover, we observed that the interaction of TIF1γ with Smad4 as well as the mono‐ubiquitination of Smad4 protein in mouse lung pericytes under TGF‐β1 treatment was also remarkably suppressed by FoxM1 overexpression (Figure 6C and D). Furthermore, the interaction of TIF1γ and Smad4 in mouse lung pericytes was greatly repressed by the knockdown of Smad3 expression using siRNAs targeting Smad3 (Figure 6E). In addition, our co‐immunoprecipitation also confirmed that FoxM1 overexpression repressed the direct binding of Smad3 with TIF1γ in moue lung pericytes (Figure 6F). Together, these results showed that FoxM1 promoted the expression of TGF‐β1 signalling through attenuating the TIF1γ‐induced Smad4 protein ubiquitination.

### 3.7 Smad3 interacts with β‐catenin, and FoxM1 binds with β‐catenin promoter to enhance fibrosis gene expression in mouse lung pericytes

**FIGURE 7:**
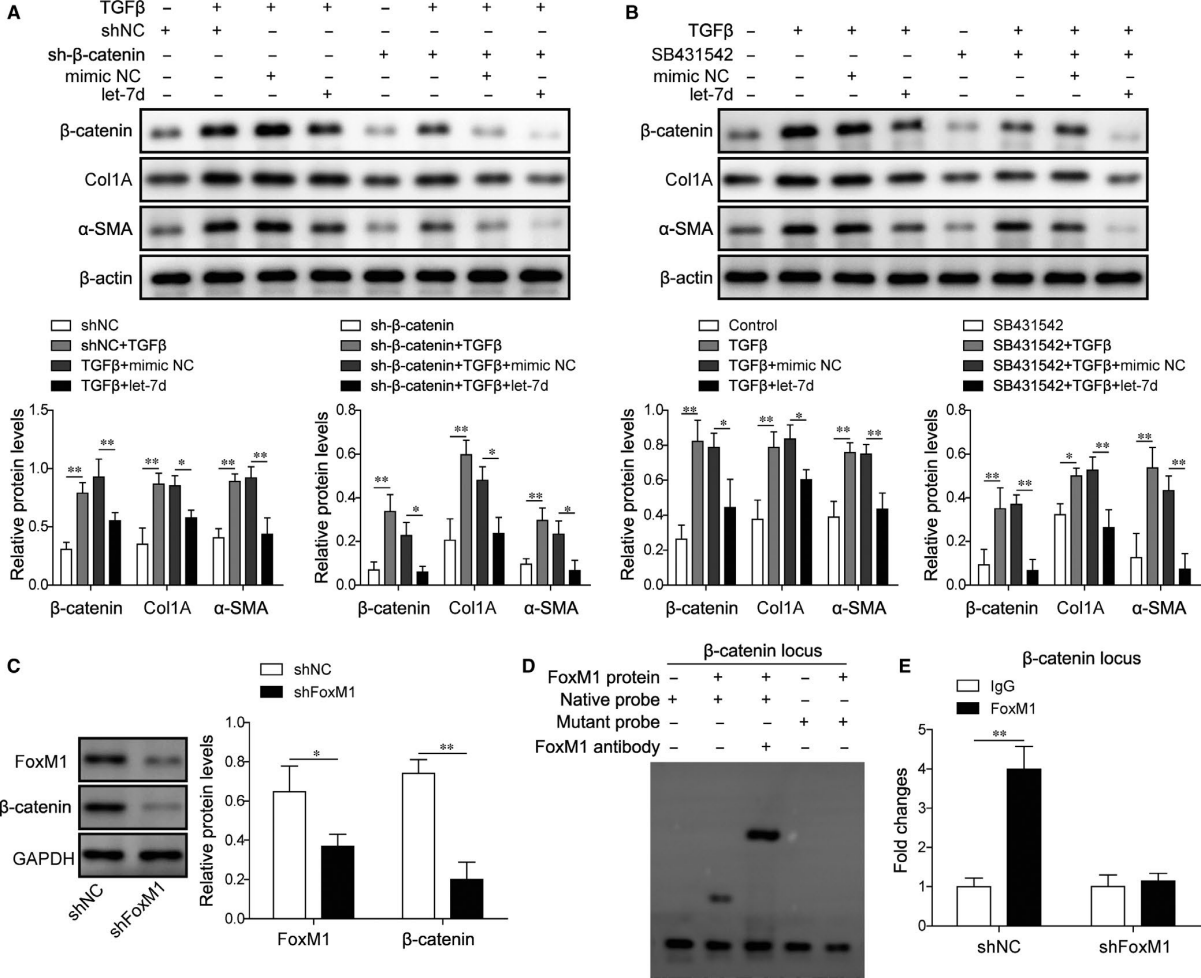
Smad3 interacts with β‐catenin and FoxM1 binds with β‐catenin promoter to enhance fibrosis gene expression in mouse lung pericytes. A and B, The expression of β‐catenin, Col1A and α‐SMA in mouse lung pericytes treated with TGF‐β1, SB431542 and shβ‐catenin. Pericytes transfected with the indicated sequences were treated with TGF‐β1 (5 ng/mL) and SB431542 (3 μmol/L) for 2 h. Fibrosis‐related gene expression was evaluated by Western blotting, using β‐actin as the internal standard. C, The abundances of FoxM1 and β‐catenin proteins in mouse lung pericytes transfected with shFoxM1. Protein levels were measured by Western blotting. D, The direct binding of FoxM1 proteins with the promoter regions of the β‐catenin gene. Association of FoxM1 with β‐catenin promoter was validated by the EMSA method. E, Influence of the FoxM1 knockdown on the association of FoxM1 protein with the promoter regions of β‐catenin. The association of FoxM1 protein with promoter sequences was measured by the ChIP method followed by PCR. TGF‐β1: transforming growth factor beta1; NC: negative control; shβ‐catenin: shRNAs targeting β‐catenin; Col1A: collagen Iα; α‐SMA: α‐smooth muscle actin; FoxM1: Forkhead box M1; GAPDH: glyceraldehyde‐3‐phosphate dehydrogenase; *P < .05; **P < .01

For more insights into the regulation of fibrosis by FoxM1 and Smad3, we finally tested the potential roles of FoxM1 and Smad3 in regulating β‐catenin gene expression as transcription factor. We showed that transfection with shRNAs targeting β‐catenin significantly repressed the expression of Col1A and α‐SMA in mouse lung pericytes under treatments with TGF‐β1 and let‐7d (Figure 7A). Also, the expression of Col1A and α‐SMA in mouse lung pericytes treated with TGF‐β1 and let‐7d was remarkably repressed by the treatment with SB431542 (Figure 7B). The knockdown of FoxM1 by shFoxM1 induced significant decrease of β‐catenin expression in mouse lung pericytes (Figure 7C). Our following EMSA assay showed that FoxM1 protein could directly bind with the promoter region of the β‐catenin (Figure 7D). Through ChIP assay combined with PCR, we showed that the association of FoxM1 protein with the promoter region of the β‐catenin was significantly repressed by the transfection with shFoxM1 in mouse lung pericytes (Figure 7E). These results showed that Smad3 interacts with β‐catenin to regulate the expression of downstream genes involved in lung fibrosis, and FoxM1 could bind with the promoter of β‐catenin to modulate fibrosis‐related gene expression in mouse lung pericytes.

## 4 DISCUSSION

The pathogenesis of pulmonary fibrosis (PF) has long been known to closely linked with the differentiation of pulmonary microvascular pericytes into myofibroblasts and abnormal deposition of extracellular matrix, which was mediated by the alterations of the TGF‐β1/Smad3/β‐catenin signalling cascade^5^, ^6^, ^7^; however, the regulatory mechanisms of this signalling axis during pericyte transformation and fibrosis remain poorly understood. In the present study, we showed that FoxM1 protein, one transcription factor previously known to substantially regulate cell proliferation and cell cycle, was highly expressed in lung fibroblasts during PF pathogenesis. On the contrary, the expression of one microRNA let‐7a was greatly down‐regulated in exosomes from broncho‐alveolar lavage fluid of mouse PF model. Our following assays proved that FoxM1 overexpression could promote the expression of Smad3, β‐catenin, Col1A and α‐SMA genes in moue lung pericytes, whereas overexpression of let‐7d then significantly repressed the expression of these genes in mouse lung pericytes. Moreover, we subsequently revealed that let‐7d performed its roles of regulating fibrosis‐related signalling pathways and gene expression in lung pericytes by directly targeting and suppressing TGFβRI. Meanwhile, FoxM1 was found in this study to enhance the nuclear retention of Smad3 proteins and prevent the TIF1γ‐mediated mono‐ubiquitination and degradation of Smad3 proteins through directly binding with Smad4 proteins. Furthermore, FoxM1 could also associate with the promoter region of the β‐catenin to regulate expression of fibrosis‐related genes in lung pericytes. These results disclosed a new TGF‐βRI/FoxM1/Smad/β‐catenin axis in lung pericytes, which could be negatively regulated by let‐7d in exosomes from pulmonary vascular endothelial cells, in mediating PF pathogenesis.

Non‐coding RNAs such as microRNAs (miRNAs), circular RNAs (circRNAs) and long non‐coding RNAs (lncRNAs) have been established as essential mediators of human diseases in the past decades.^27^, ^28^ Among them, microRNAs serve as potent regulator of gene expression via directly binding with the 3’ UTR region of functional genes, thus being reported to be widely involved in pathogenic processes of various human diseases including idiopathic pulmonary fibrosis (IPF).^29^, ^30^, ^31^, ^32^ On the other hand, microRNAs have also been characterized as one major group of cargos that could be transferred by exosomes to exert physiological and pathogenic functions in tissues away from its original sites.^21^, ^22^, ^23^ However, litter is recently known about the roles of microRNAs encapsulated in exosomes secreted by pulmonary vascular endothelial cells in regulating lung pericyte fibrosis. In this study, we found that the expression of let‐7d, a microRNA previously known to suppress TGF‐β1‐induced renal fibrogenesis,^25^ was significantly reduced in exosomes from mouse PF model. Importantly, the expression of Smad3 and β‐catenin in mouse lung pericytes, as well as Col1A and α‐SMA involved in fibrosis, was significantly repressed by overexpression of let‐7d, but elevated by treatment with these low let‐7d isolated from model mice compared with exosomes from normal mice. More importantly, we proved here that let‐7d could directly target the TGFβRI in mouse pericytes, which indicated that the modulation of TGFβRI expression by non‐coding RNAs might serve as a promising strategy to prevent PF pathogenesis.

FoxM1 (Forkhead box M1) was previously reported as an essential regulator of the Wnt/β‐catenin signalling pathway through directly binding with β‐catenin protein to retain β‐catenin in nucleus and promoting the β‐catenin transcription.^13^, ^14^, ^15^ Also, FoxM1 could enhance the TGF‐β1/Smad3 signalling in cancer cells by stabilizing Smad3/Smad4 complex and inhibiting Smad4 protein degradation induced by TIF1γ.^16^ Here, in the present study, we showed that FoxM1 expression was highly elevated in the lung fibroblasts in mouse model and human tissues with PF. Moreover, the expression of FoxM1 gene in mouse lung pericytes could be enhanced by TGF‐β1 treatment and let‐7d inhibitor, but effectively down‐regulated by let‐7d mimics. Of significance, we showed here that FoxM1 overexpression in mouse lung pericytes significantly elevated the expression of Smad3, β‐catenin, Col1A and α‐SMA, whose expression could also be repressed by FoxM1 knockdown in mouse lung pericytes under TGF‐β1 treatment. These results convincingly supported the roles of FoxM1 as a key positive mediator of TGF‐β1‐induced lung pericyte fibrosis during PF pathogenesis, which is consistent with previous report showing the association of FoxM1 with transformation of lung pericytes into myofibroblasts.^17^ In terms of molecular mechanisms, we showed in this study that the FoxM1 could directly associate with the Smad3 protein, which resulted in greatly increased Smad3 nuclear retention and promoted expression of downstream genes. Furthermore, we also showed that the mono‐ubiquitination and degradation of Smad4, which was induced by TIF1γ, could be inhibited by the binding of FoxM1 protein with Smad3 protein. Together, our above‐mentioned findings in this study indicated that the regulation of Smad3/4 protein complex stability and Smad3 nuclear retention might be a prevalent molecular mechanism of FoxM1 functioning in distinct physiological and pathogenic contexts.

As introduced above, the regulation of β‐catenin transcription and nuclear distribution by FoxM1 protein was critically associated with the TGF‐β1 signalling cascade.^13^, ^14^, ^15^ Previous report proved that β‐catenin serves as one important co‐factor of the TCF (T‐cell factor) transcription factors, which could mediate various biological processes by promoting the expression of a multitude of downstream functional genes such as cyclin‐D1, c‐myc and survivin.^33^ The activation of β‐catenin signalling has been suggested as a common feature of lung fibrosis, because of its potent roles of promoting pulmonary fibroblast proliferation and migration.^33^, ^34^ In this study, we showed the expression of β‐catenin in mouse lung pericytes was significantly increased by TGF‐β1 treatment, let‐7d inhibitor and FoxM1 overexpression, but greatly repressed by let‐7d mimic, TGFβRI inhibitor and FoxM1 knockdown. Also, β‐catenin knockdown with shRNAs caused significant decreases of Col1A and α‐SMA expression in mouse lung pericytes under TGF‐β1 treatment. Importantly, we finally showed that FoxM1 proteins could be directly associated with promoter region of the β‐catenin in mouse lung pericytes by both EMSA and ChIP‐PCR assays, which validated the roles of β‐catenin in PF development, as well as the diversity of molecular mechanisms underlying FoxM1 and β‐catenin‐mediated lung pericyte fibrosis.

## 5 CONCLUSIONS

In summary, we unveiled in this study that the microRNA let‐7d, whose expression was suppressed in exosomes derived from pulmonary vascular endothelial cells, could target and repress the TGFβRI to modulate the downstream FoxM1/Smad/β‐catenin signalling axis and lung pericyte fibrosis. FoxM1 protein could enhance the nuclear retention of Smad3 protein and stabilize Smad3/4 protein complex to promote the expression of fibrotic gene expression in lung pericytes by directly binding with Smad3 protein. These results provided novel insights into the pathogenesis of PF mediated by the TGF‐β1/Smad and Wnt/β‐catenin signalling cascades, which might serve as a basis for developing new drugs preventing PF development.

## CONFLICT OF INTEREST

The authors declare that there is no conflict of interest.

## AUTHOR CONTRIBUTIONS

**Han Xie:** Resources (equal). **Yuan‐Mei Gao:** Writing‐review & editing (equal). **Yong‐Chang Zhang:** Resources (equal). **Ming‐Wang Jia:** Writing‐original draft (equal). **Fang Peng:** Resources (equal). **Qing‐He Meng:** Writing‐review & editing (equal). **Yi‐Chun Wang:** Writing‐review & editing (equal).

## ETHICAL APPROVAL

The research was approved by the Medical Ethics Committee of the Third Affiliated Hospital of Guangzhou Medical University, and written consents were assigned by each participant before the surgery.

## Data Availability

All data generated or analyzed during this study are included in this published article.

## References

[1] Glasser SW , Hagood JS , Wong S , Taype CA , Madala SK , Hardie WD . Mechanisms of lung fibrosis resolution. Am J Pathol. 2016;186:1066‐1077. 27021937 10.1016/j.ajpath.2016.01.018 PMC4861766

[2] Richeldi L , Collard HR , Jones MG . Idiopathic pulmonary fibrosis. Lancet. 2017;389:1941‐1952. 28365056 10.1016/S0140-6736(17)30866-8

[3] Scotton CJ , Chambers RC . Molecular targets in pulmonary fibrosis: the myofibroblast in focus. Chest. 2007;132:1311‐1321. 17934117 10.1378/chest.06-2568

[4] Yoshida T , Ohnuma A , Horiuchi H , Harada T . Pulmonary fibrosis in response to environmental cues and molecular targets involved in its pathogenesis. J Toxicol Pathol. 2011;24:9‐24. 22272040 10.1293/tox.24.9 PMC3234628

[5] Harrell CR , Simovic Markovic B , Fellabaum C , Arsenijevic A , Djonov V , Volarevic V . Molecular mechanisms underlying therapeutic potential of pericytes. J Biomed Sci. 2018;25:21. 29519245 10.1186/s12929-018-0423-7 PMC5844098

[6] Sava P , Ramanathan A , Dobronyi A , Peng X , Gonzalez AL . Human pericytes adopt myofibroblast properties in the microenvironment of the IPF lung. JCI Insight. 2017;2(24):e96352 10.1172/jci.insight.96352 PMC5752282 29263297

[7] Sun W , Tang H , Gao L , et al. Mechanisms of pulmonary fibrosis induced by core fucosylation in pericytes. Int J Biochem Cell Biol. 2017;88:44‐54. 28483669 10.1016/j.biocel.2017.05.010

[8] Li J , Wang G , Sun X . Transforming growth factor beta regulates beta‐catenin expression in lung fibroblast through NF‐kappaB dependent pathway. Int J Mol Med. 2014;34:1219‐1224. 25175023 10.3892/ijmm.2014.1916 PMC4199410

[9] Xu L , Cui W‐H , Zhou W‐C , et al. Activation of Wnt/β‐catenin signalling is required for TGF‐β/Smad2/3 signalling during myofibroblast proliferation. J Cell Mol Med. 2017;21:1545‐1554. 28244647 10.1111/jcmm.13085 PMC5542906

[10] Halasi M , Gartel AL . A novel mode of FoxM1 regulation: positive auto‐regulatory loop. Cell Cycle. 2009;8:1966‐1967. 19411834 10.4161/cc.8.12.8708

[11] Laura B , Stefania Z , de Moraes GN , Eric W‐F . FOXM1: A key oncofoetal transcription factor in health and disease. Semin Cancer Biol. 2014;29:8. 10.1016/j.semcancer.2014.07.008 25068996

[12] Koo CY , Muir KW , Lam EW . FOXM1: From cancer initiation to progression and treatment. Biochim Biophys Acta. 2012;1819:28‐37. 21978825 10.1016/j.bbagrm.2011.09.004

[13] Bowman A , Nusse R . Location, location, location: FoxM1 mediates beta‐catenin nuclear translocation and promotes glioma tumorigenesis. Cancer Cell. 2011;20:415‐416. 22014565 10.1016/j.ccr.2011.10.003

[14] Zhang N , Wei P , Gong A , et al. FoxM1 promotes beta‐catenin nuclear localization and controls Wnt target‐gene expression and glioma tumorigenesis. Cancer Cell. 2011;20:427‐442. 22014570 10.1016/j.ccr.2011.08.016 PMC3199318

[15] Mirza MK , Sun Y , Zhao YD , et al. FoxM1 regulates re‐annealing of endothelial adherens junctions through transcriptional control of β‐catenin expression. J Exp Med. 2010;207:1675‐1685. 20660612 10.1084/jem.20091857 PMC2916140

[16] Xue J , Lin X , Chiu W‐T , et al. Sustained activation of SMAD3/SMAD4 by FOXM1 promotes TGF‐β–dependent cancer metastasis. Journal of Clinical Investigation. 2014;124:564‐579. 10.1172/JCI71104 PMC3904622 24382352

[17] Penke LR , Speth JM , Dommeti VL , White ES , Bergin IL , Peters‐Golden M . FOXM1 is a critical driver of lung fibroblast activation and fibrogenesis. J Clin Invest. 2018;128:2389‐2405. 29733296 10.1172/JCI87631 PMC5983327

[18] Meng W , Hao Y , He C , Li L , Zhu G . Exosome‐orchestrated hypoxic tumor microenvironment. Mol Cancer. 2019;18:57. 30925935 10.1186/s12943-019-0982-6 PMC6441221

[19] Wortzel I , Dror S , Kenific CM , Lyden D . Exosome‐mediated metastasis: communication from a distance. Dev Cell. 2019;49:347‐360. 31063754 10.1016/j.devcel.2019.04.011

[20] Butler JS . The yin and yang of the exosome. Trends Cell Biol. 2002;12:90‐96. 11849973 10.1016/s0962-8924(01)02225-5

[21] Kita S , Maeda N , Shimomura I . Interorgan communication by exosomes, adipose tissue, and adiponectin in metabolic syndrome. J Clin Invest. 2019;129:4041‐4049. 31483293 10.1172/JCI129193 PMC6763291

[22] Vyas N , Dhawan J . Exosomes: mobile platforms for targeted and synergistic signaling across cell boundaries. Cell Mol Life Sci. 2017;74:1567‐1576. 27826642 10.1007/s00018-016-2413-9 PMC11107587

[23] Singh R , Pochampally R , Watabe K , Lu Z , Mo YY . Exosome‐mediated transfer of miR‐10b promotes cell invasion in breast cancer. Mol Cancer. 2014;13:256. 25428807 10.1186/1476-4598-13-256 PMC4258287

[24] Elliot S , Periera‐Simon S , Xia X , et al. MicroRNA let‐7 downregulates ligand‐independent estrogen receptor‐mediated male‐predominant pulmonary fibrosis. Am J Respir Crit Care Med. 2019;200:1246‐1257. 31291549 10.1164/rccm.201903-0508OC PMC6857483

[25] Wang Y , Le Y , Xue JY , Zheng ZJ , Xue YM . Let‐7d miRNA prevents TGF‐beta1‐induced EMT and renal fibrogenesis through regulation of HMGA2 expression. Biochem Biophys Res Comm. 2016;479:676‐682. 27693697 10.1016/j.bbrc.2016.09.154

[26] Carnino JM , Lee H , Jin Y . Isolation and characterization of extracellular vesicles from Broncho‐alveolar lavage fluid: a review and comparison of different methods. Respir Res. 2019;20:240. 31666080 10.1186/s12931-019-1210-z PMC6822481

[27] de Almeida RA , Fraczek MG , Parker S , Delneri D , O'Keefe RT . Non‐coding RNAs and disease: the classical ncRNAs make a comeback. Biochem Soc Trans. 2016;44:1073‐1078. 27528754 10.1042/BST20160089 PMC6042638

[28] Nothnick WB . Non‐coding RNAs in uterine development, function and disease. Adv Exp Med Biol. 2016;886:171‐189. 26659492 10.1007/978-94-017-7417-8_9 PMC4878440

[29] Chen X , Xie D , Zhao Q , You ZH . MicroRNAs and complex diseases: from experimental results to computational models. Brief Bioinform. 2017;18:558. 27345524 10.1093/bib/bbw060 PMC5862301

[30] Li H , Zhao X , Shan H , Liang H . MicroRNAs in idiopathic pulmonary fibrosis: involvement in pathogenesis and potential use in diagnosis and therapeutics. Acta Pharm Sin B. 2016;6:531‐539. 27818919 10.1016/j.apsb.2016.06.010 PMC5071633

[31] Mizuno K , Mataki H , Seki N , Kumamoto T , Kamikawaji K , Inoue H . MicroRNAs in non‐small cell lung cancer and idiopathic pulmonary fibrosis. J Hum Genet. 2017;62:57‐65. 27488441 10.1038/jhg.2016.98

[32] Quinlan S , Kenny A , Medina M , Engel T , Jimenez‐Mateos EM . MicroRNAs in neurodegenerative diseases. Int Rev Cell Mol Biol. 2017;334:309. 28838542 10.1016/bs.ircmb.2017.04.002

[33] Lee JM , Yang J , Newell P , et al. beta‐Catenin signaling in hepatocellular cancer: Implications in inflammation, fibrosis, and proliferation. Cancer Lett. 2014;343:90‐97. 24071572 10.1016/j.canlet.2013.09.020 PMC3874258

[34] Lam AP , Flozak AS , Russell S , et al. Nuclear beta‐catenin is increased in systemic sclerosis pulmonary fibrosis and promotes lung fibroblast migration and proliferation. Am J Respir Cell Mol Biol. 2011;45:915‐922. 21454805 10.1165/rcmb.2010-0113OC PMC3262680

